# Complete Reference Genome Sequence of the Clinical Neisseria gonorrhoeae Strain H035, with Resistance to the Novel Antimicrobial Zoliflodacin, Identified in Japan in 2000

**DOI:** 10.1128/mra.01130-22

**Published:** 2023-02-28

**Authors:** Daniel Golparian, Susanne Jacobsson, Makoto Ohnishi, Magnus Unemo

**Affiliations:** a World Health Organization Collaborating Centre for Gonorrhoea and Other Sexually Transmitted Infections, Department of Laboratory Medicine, Microbiology, Faculty of Medicine and Health, Örebro University, Örebro, Sweden; b Department of Bacteriology I, National Institute of Infectious Diseases, Tokyo, Japan; c Institute for Global Health, University College London, London, United Kingdom; Loyola University Chicago

## Abstract

Zoliflodacin is a promising novel antimicrobial in clinical development for treatment of gonorrhea; currently, it is in a global phase 3 randomized controlled clinical trial. High activity against global Neisseria gonorrhoeae strains has been shown. We present the complete reference genome of the zoliflodacin-resistant strain H035, which was identified in Japan in 2000.

## ANNOUNCEMENT

Neisseria gonorrhoeae has developed resistance to all antimicrobials recommended for gonorrhea treatment (1, 2). New antimicrobials for gonorrhea treatment are needed, as emphasized by the WHO (3). One novel drug in clinical development is zoliflodacin, which was exceedingly promising *in vitro* (4, 5) and currently is in a global phase 3 randomized controlled clinical trial (6). Recently, screening of all available gonococcal genomes for zoliflodacin resistance-associated mutations, combined with zoliflodacin testing, confirmed high global susceptibility rates (7). Only one strain carried a zoliflodacin resistance-associated mutation (GyrB D429V) (7). We report the complete reference genome of this zoliflodacin-resistant clinical strain (H035), which was isolated in Japan in 2000, to provide a genomic reference for future zoliflodacin studies.

H035 was cultured on modified Thayer-Martin agar at 36°C ± 1°C in 5% CO_2_ for 24 h from a sample from the urethra of a 33-year-old Japanese male patient in 2000 (7). Prior to DNA extraction using the Nanobind CBB Big DNA kit (Circulomics), H035 was cultured on chocolate agar at 36°C ± 1°C in 5% CO_2_ for 24 h. Nanopore (Oxford Nanopore Technologies [ONT]) sequencing was performed without DNA shearing, size selection, or fragmentation, and short fragments (<3 kb) were excluded using long fragment buffer (Nanopore). The Nanopore sequencing library was prepared using the duplex Nanopore Q20+ chemistry (SQK-LSK112) and sequenced on an Mk1C system for 24 h with a R10.4 flow cell (FLO-MIN112). Bases were called using the ONT guppy duplex pipeline v1.0.0 with the super high accuracy (SUP) option, which generated 34,737 duplex reads with Phred quality scores of >Q30 (read *N*_50_, 9,652 bp). Adapters were trimmed using Porechop v0.2.4 (https://github.com/rrwick/Porechop). An Illumina DNA preparation kit was used for Illumina paired-end library preparation, according to the manufacturer’s recommendations, and a NextSeq 550 system (Illumina) was used for paired-end short-read sequencing (read *N*_50_, 100 bp), generating 4,716,356 reads (average coverage depth, 234×). DNA was sheared using enzymatic fragmentation (average fragment size, 469 bp) with magnetic beads, according to Illumina protocols, and sequenced without size selection. All reads were quality controlled using CLC Genomics Workbench v22.0.2, and low-quality bases (scores of <Q30) were removed. Kraken v1.1.1 (http://ccb.jhu.edu/software/kraken) was used for species verification. Hybrid assembly using Nanopore duplex reads and Illumina paired-end reads was performed using Unicycler v0.4.7 (8), which was also utilized to rotate the single contig to start with DnaA (UniProtKB accession number Q9JXS7) and the circularized cryptic plasmid (pJD1) to start with the replication initiator protein (UniProtKB accession number Q50999). Default parameters were used for all software unless specified.

The complete H035 chromosome was 2,167,942 bp (G+C content, 52.6%). The Prokaryotic Genome Annotation Pipeline (PGAP) v6.1 (9) annotated 2,225 coding sequences, 56 tRNAs, and 12 rRNAs. Additionally, H035 harbored a cryptic plasmid (4,197 bp) with 9 coding sequences. H035 was resistant to zoliflodacin (MIC, 8 mg/L [GyrB D429V]) in agar dilution (7), ciprofloxacin (2 mg/L [GyrA S91F and ParC S87R/S88P]), sulfonamides, benzylpenicillin, and tetracycline and showed decreased susceptibility to ceftriaxone and cefixime (0.125 mg/L [mosaic *penA*-10.001, MtrR G45D, and *mtrR* promoter A57 deletion]). Fortunately, the zoliflodacin-resistant strain H035 has not clonally expanded and the zoliflodacin resistance-associated *gyrB* mutation has not been disseminated to other gonococcal strains (7).

Isolate H035 was cultured using routine diagnostic methods. No ethical approval was needed to publish the isolate without any patient-identifying data.

### Data availability.

The complete sequences of H035 were deposited in GenBank under accession numbers CP107223.1 (chromosome) and CP107224.1 (cryptic plasmid). The raw reads are available through the NCBI Sequence Read Archive (SRA) under accession numbers SRX17825415 (Illumina) and SRX17825416 (Nanopore). The project summary can be found under BioProject accession number PRJNA887939.

## References

[1] Unemo M, Seifert HS, Hook EW, III, Hawkes S, Ndowa F, Dillon JR. 2019. Gonorrhoea. Nat Rev Dis Primers 5:79. doi:10.1038/s41572-019-0128-6. 31754194

[2] Unemo M, Lahra MM, Escher M, Eremin S, Cole MJ, Galarza P, Ndowa F, Martin I, Dillon JR, Galas M, Ramon-Pardo P, Weinstock H, Wi T. 2021. WHO global antimicrobial resistance surveillance for *Neisseria gonorrhoeae* 2017–18: a retrospective observational study. Lancet Microbe 2:e627–e636. doi:10.1016/S2666-5247(21)00171-3. 35544082

[3] World Health Organization. 27 February 2017. WHO publishes list of bacteria for which new antibiotics are urgently needed. https://www.who.int/en/news-room/detail/27-02-2017-who-publishes-list-of-bacteria-for-which-new-antibiotics-are-urgently-needed.

[4] Unemo M, Ahlstrand J, Sánchez-Busó L, Day M, Aanensen D, Golparian D, Jacobsson S, Cole MJ, European Collaborative Group. 2021. High susceptibility to zoliflodacin and conserved target (GyrB) for zoliflodacin among 1209 consecutive clinical *Neisseria gonorrhoeae* isolates from 25 European countries, 2018. J Antimicrob Chemother 76:1221–1228. doi:10.1093/jac/dkab024. 33564854

[5] Jacobsson S, Golparian D, Oxelbark J, Franceschi F, Brown D, Louie A, Drusano G, Unemo M. 2022. Pharmacodynamic evaluation of zoliflodacin treatment of *Neisseria gonorrhoeae* strains with amino acid substitutions in the zoliflodacin target GyrB using a dynamic hollow fiber infection model. Front Pharmacol 13:874176. doi:10.3389/fphar.2022.874176. 35496288 PMC9046595

[6] Droal A, Luckey A. 2021. Zoliflodacin in uncomplicated gonorrhoea. ClinicalTrials.gov registration number NCT03959527. https://www.clinicaltrials.gov/ct2/show/NCT03959527.

[7] Golparian D, Jacobsson S, Sánchez-Busó L, Bazzo ML, Lan PT, Galarza P, Ohnishi M, Unemo M. 2022. GyrB in silico mining in 27151 global gonococcal genomes from 1928–2021 combined with zoliflodacin in vitro testing of 71 international gonococcal isolates with different GyrB, ParC and ParE substitutions confirms high susceptibility. J Antimicrob Chemother 78:150–154. doi:10.1093/jac/dkac366. 36308328

[8] Wick RR, Judd LM, Gorrie CL, Holt KE. 2017. Unicycler: resolving bacterial genome assemblies from short and long sequencing reads. PLoS Comput Biol 13:e1005595. doi:10.1371/journal.pcbi.1005595. 28594827 PMC5481147

[9] Tatusova T, DiCuccio M, Badretdin A, Chetvernin V, Nawrocki EP, Zaslavsky L, Lomsadze A, Pruitt KD, Borodovsky M, Ostell J. 2016. NCBI Prokaryotic Genome Annotation Pipeline. Nucleic Acids Res 44:6614–6624. doi:10.1093/nar/gkw569. 27342282 PMC5001611

